# After COVID-19: Rethinking Fiscal Rules in Europe

**DOI:** 10.1007/s10272-020-0914-0

**Published:** 2020-10-24

**Authors:** Forum Introduction

## Abstract

Following the COVID-19 outbreak in Europe this spring and subsequent measures to contain the pandemic, the European Commission drastically revised its economic and fiscal forecasts. The Summer 2020 Economic Forecast projects that the euro area economy will contract by 8.7% in 2020. The coronavirus crisis is expected to push the general government deficit to about 8.5% of GDP this year. Even in an optimistic V-shaped recovery scenario with a GDP growth rate of 6.1% in 2021 due to the temporary nature of lockdown measures taken in 2020, the headline deficit is expected to decrease to about 3.5% of GDP. Furthermore, both the downturn and the rebound of economic activity are expected to be asymmetric across member states, exposing entrenched divergences. The recent outlook highlights the problem of pro-cyclical revisions of potential output and output gap estimates. Some economists warn that the current fiscal framework may lead to pro-cyclical and thus destabilising fiscal policies, a problem encountered in Southern Europe during the European sovereign debt crisis that has implications for the entire European Union. In order to avoid repeating past mistakes, the debate on how to reform European fiscal policy should be settled before the rules are re-enacted when the coronavirus crisis has passed.

